# Quorum Sensing Signaling Alters Virulence Potential and Population Dynamics in Complex Microbiome-Host Interactomes

**DOI:** 10.3389/fmicb.2019.02131

**Published:** 2019-09-11

**Authors:** F. Jerry Reen, José A. Gutiérrez-Barranquero, Ronan R. McCarthy, David F. Woods, Sara Scarciglia, Claire Adams, Kristian Fog Nielsen, Lone Gram, Fergal O’Gara

**Affiliations:** ^1^BIOMERIT Research Centre, School of Microbiology, University College Cork, Cork, Ireland; ^2^School of Microbiology, University College Cork, Cork, Ireland; ^3^Department of Biotechnology and Biomedicine, Technical University of Denmark, Lyngby, Denmark; ^4^Telethon Kids Institute, Perth Children’s Hospital, Perth, WA, Australia; ^5^School of Pharmacy and Biomedical Sciences, Curtin Health Innovation Research Institute, Curtin University, Perth, WA, Australia

**Keywords:** quorum sensing (QS), microbiome, marine sponge-associated bacteria, cell–cell communication, acyl homoserine lactone (AHL)

## Abstract

Despite the discovery of the first *N*-acyl homoserine lactone (AHL) based quorum sensing (QS) in the marine environment, relatively little is known about the abundance, nature and diversity of AHL QS systems in this diverse ecosystem. Establishing the prevalence and diversity of AHL QS systems and how they may influence population dynamics within the marine ecosystem, may give a greater insight into the evolution of AHLs as signaling molecules in this important and largely unexplored niche. Microbiome profiling of *Stelletta normani* and BD1268 sponge samples identified several potential QS active genera. Subsequent biosensor-based screening of a library of 650 marine sponge bacterial isolates identified 10 isolates that could activate at least one of three AHL biosensor strains. Each was further validated and profiled by Ultra-High Performance Liquid Chromatography Mass Spectrometry, with AHLs being detected in 8 out of 10 isolate extracts. Co-culture of QS active isolates with *S. normani* marine sponge samples led to the isolation of genera such as *Pseudomonas* and *Paenibacillus*, both of which were low abundance in the *S. normani* microbiome. Surprisingly however, addition of AHLs to isolates harvested following co-culture did not measurably affect either growth or biofilm of these strains. Addition of supernatants from QS active strains did however impact significantly on biofilm formation of the marine *Bacillus* sp. CH8a sporeforming strain suggesting a role for QS systems in moderating the microbe-microbe interaction in marine sponges. Genome sequencing and phylogenetic analysis of a QS positive *Psychrobacter* isolate identified several QS associated systems, although no classical QS synthase gene was identified. The stark contrast between the biodiverse sponge microbiome and the relatively limited diversity that was observed on standard culture media, even in the presence of QS active compounds, serves to underscore the extent of diversity that remains to be brought into culture.

## Introduction

The marine ecosystem is considered to be an underexplored resource for the study of bacterial interactions within eukaryotic hosts. Despite a number of well-studied examples of bacterial interactions within marine hosts such as the density dependent production of luminescence by *Aliivibrio fischeri* within the light organ of *Euprymna scolopes*, relatively little is known about the interactions that occur within marine microbial communities (Hmelo, 2017). This is particularly true in the case of the ancient invertebrate, the marine sponge. Marine sponges are sessile filter feeders that consume bacteria and other marine matter (Taylor et al., 2007). Bacteria can inhabit the mesophyll matrix of these invertebrates with almost 60% of the biomass of a marine sponge being comprised of bacterial endosymbionts (Wang, 2006). This symbiotic relationship is mutually beneficial whereby bacteria are provided with a sheltered nutrient rich environment and the marine sponges acquire limiting nutrients from the microflora (Mohamed et al., 2008; Blunt et al., 2009; Mayer et al., 2010, 2011). Within the dense polymicrobial environment of a marine sponge, bacteria can engage in a form of chemical communication termed quorum sensing (QS) (Taylor et al., 2004a; Diggle et al., 2007; Hmelo, 2017). Several classes of QS signaling system are known, with autoinducer peptides favored by gram positive bacteria while *N*-acyl homoserine lactones (AHLs) predominate within gram negative bacteria (Whiteley et al., 2017). AHLs are capable of activating an autoinducing transcriptional regulator which controls the transcription of target genes involved in a wide variety of cellular processes including the production of virulence determinants (Diggle et al., 2007). There are relatively few studies on the prevalence of functional AHL based QS systems within microorganisms inhabiting marine sponges (Taylor et al., 2004b; Mohamed et al., 2008; Cuadrado-Silva et al., 2013; Britstein et al., 2018). A number of studies have focused on the identification of homologs of genes associated with QS pathways (Zan et al., 2011). However, sequence based approaches provide limited information on the functionality of these homologous systems, which for the most part remains to be determined. This homology-based approach is also limited by the lack of nucleotide sequence homology among AHL synthases and AHL responsive transcriptional regulators (Steindler and Venturi, 2007). More recently, screening of marine sponges for AHL signals has revealed a rich diversity likely encoded by the microbial communities residing in those sponges (Britstein et al., 2018). Given the difficulties faced in bringing marine sponge biodiversity into culture, it is intriguing to speculate that these signals may play a role in moderating the dynamics of the microbial communities within which they operate.

To gain more insight into the relevance of AHL based QS systems within the microbiota inhabiting marine sponges, bacterial sponge isolates were screened for the production of AHLs using classical AHL reporter strains. A total of 10 QS producing isolates were identified and characterized for AHL production. Co-culture of QS positive isolates with marine sponge samples resulted in increased culturable plate diversity from these communities, although no new genera were identified. While addition of AHLs alone did not influence growth or biofilm in the marine sponge isolates, supernatants from several QS positive isolates suppressed biofilm formation in the marine sponge *Bacillus* sp. CH8a sporeforming strain. This suggests that the anti-biofilm activity of the QS active supernatants may be mediated downstream of intact QS signaling systems in the producing isolates. Genome sequencing of a QS positive *Psychrobacter* sp. isolate identified in this study revealed the presence of LuxR DNA binding domains. However, there was no evidence of a LuxR autoinducer domain or an AHL synthase domain in this or any other sequenced *Psychrobacter* genome. Further establishing the prevalence, structure and diversity of AHL based QS systems will give a better understanding of the role of AHL signaling in the marine ecosystem, potentially unlocking some of the natural biodiversity encoded therein.

## Materials and Methods

### Sponge Collection

Bacteria had previously been isolated from sponge genera including *Hexactinellida*, *Stelletta*, *Lissodendoryx*, *Poecillastra*, *Inflatella*. These sponges were collected using a remote operated vehicle on board the Celtic Explorer research vessel, 300 nautical miles off the west coast of Ireland as part of the marine biodiscovery cruise, May 2010. Sponge samples from the *Amphilectus* genus were collected in Gurraig Sound Kilkieran Bay, Galway (Kennedy et al., 2008). Whole sponge samples were rinsed with sterile artificial sea water (ASW, 3.33% (w/v) artificial sea salts, Instant Ocean) and immediately frozen at −80°C on board the ship until further processing. A sample of sponge tissue (1 g) was homogenized by grinding with a sterile porcelain pestle and mortar in 9 ml of sterile ASW. The sponge homogenate was subsequently serially diluted in ASW to 10^–5^ and 100 μl aliquots of the different dilutions were plated onto Marine agar (MA) (Difco, United Kingdom) and SYP-SW Agar (1% (w/v) starch, 0.4% (w/v) yeast extract, 0.2% (w/v) peptone and 3.33% (w/v) artificial sea salts, 1.5% (w/v) agar. Distinct morphologies were collected to form a library of approximately 650 isolates for subsequent screening and characterization.

### Sponge Microbiome Profiling

As above, two independent samples of each sponge tissue (1 g) were homogenized by grinding with a sterile porcelain pestle and mortar in 10 ml of sterile PBS. DNA was extracted from 100 μl of these samples using the MoBio DNA extraction kit (MoBio) as per manufacturer’s instructions. The extracted gDNA was used as a template to amplify the v3-v5 region of the 16S rRNA gene, these amplicons were sequenced to 2 × 300 bp on a Next Gen Illumina MiSeq (V3) platform. Prior to the microbiome analysis, raw reads were demultiplexed based on inline-barcode sequences. The reads were processed using Minimum Entropy Decomposition (Eren et al., 2015). To assign taxonomic information to each Operational Taxonomic Unit (OTU), BLAST alignments of representative sequences to the NCBI database were performed. Further processing of OTUs was performed using the QIIME software package (version 1.8.0^1^). Microbiome data has been uploaded on the NCBI Sequence Read Archive (SRA) database (BioProject No. PRJNA555824).

### Quorum Sensing Biosensor Assay

The following reporter strains were used to identify AHL production. Short chain AHLs were detected using the *Serratia marcescens* SP19 (Poulter et al., 2010). Upon production of short chain AHLs *S. marcescens* SP19 produces a red pigment, prodigiosin. *S. marcescens* SP19 is an AHL deficient mutant that only produces prodigiosin in response exogenous AHLs. Marine strains were cultured on Marine Broth (Difco, United Kingdom) supplemented with agar (1.5% w/v) for 72 h at 23°C. They were then overlaid with soft LB agar (0.1% agar) inoculated with *S. marcescens* SP19 at an OD_600 nm_ of 0.5. Overlaid plates were incubated at 30°C overnight. QS was identified by prodigiosin production. As a positive control, 20 μM C4-HSL (Sigma, United Kingdom) was used. C4-C8 chain AHLs were detected using *Chromobacterium violaceum* CV026. *C. violaceum* CV026 produces a purple compound called violacein in a QS dependent manner. *C*. *violaceum* CV026 contains a transposon in its indigenous AHL synthase gene thus it only produces violacein in response to exogenous AHLs (McClean et al., 1997). Marine strains were cultured on Marine Agar (Difco, United Kingdom) for 72 h at 30°C. They were then overlaid with soft LB agar (0.1% agar) inoculated with *C*. *violaceum* CV026 at an OD_600__nm_ of 0.5. Overlaid plates were incubated at 30°C overnight. QS was identified by violacein production. 20 μM C8-HSL (Sigma, United Kingdom) was used as a positive control. The broad range *Agrobacterium tumefaciens* NTL4 biosensor was used for the detection of longer chain AHLs. It contains a plasmid pZLR4 carrying a *traG:lacZ* reporter fusion (Farrand et al., 1996; Yin et al., 2012). In response to exogenous AHLs the *lacZ* gene is transcribed resulting in the degradation of 5-bromo-4-chloro-3-indolyl-β-D-galactopyranoside in the media. The previously described plating protocol was observed except soft LB was supplemented with 50 μg/ml 5-bromo-4-chloro-3-indolyl-β-D-galactopyranoside. QS was identified by the breakdown of 5-bromo-4-chloro-3-indolyl-β-D-galactopyranoside resulting in a blue color. A concentration of 20 μM C10-HSL (Sigma, United Kingdom) was used as a positive control.

### Strain Identification

Genomic DNA from bacterial isolates was extracted using the MoBio UltraClean DNA extraction kit (MoBio, United States) following manufacturers’ guidelines. Strains were identified by PCR amplification of 16S rRNA genes which was carried out as described (Chan et al., 2010) using the universal primer pairs 27F (5′-AGAGTTTGATCMTGGCTCAG-3′) and 1525R (5′-AAGGAGGTGWTCCARCC-3′). PCR products were sequenced by MWG Eurofins, United Kingdom. Sequence identification was performed using BlastN and all sequences were submitted to the NCBI database (Accession numbers: MN209943-MN209952).

### Extraction and TLC Analysis of Culture Supernatants

Extracts for thin layer chromatography (TLC) and ultra-high performance liquid chromatography-high resolution mass spectrometry (UHPLC-HRMS) were prepared from 200 ml cultures in Marine Broth (Difco, United Kingdom) that had been incubated for 72 h at 30°C and 180 rpm. Bacterial cells were pelleted by centrifugation at 4,000 rpm for 7 min and the supernatant was filter sterilized using a Nalgene^®^ vacuum filtration system (0.2 μm, Sigma-Aldrich^®^, Germany). The supernatant was incubated with 1:1 volume of acidified ethyl acetate (1% formic acid) for 10 min at RT shaking at 180 rpm. The ethyl acetate phase was taken and dried using rotary evaporation. Residues were resuspended in 1 ml ethyl acetate and stored at −20°C. Ethyl acetate extracts were tested for QS activity by spotting 20 μL on C18 reverse phase TLC plates (20 cm × 20 cm TLC aluminum plates, Millipore, United Kingdom) with a methanol:water 7:3 (v/v) mobile phase. Once dried, TLC plates were overlaid with 25 ml of soft LB agar (0.1% agar) inoculated with the biosensor at an OD_600__nm_ of 0.5. Following confirmation of extract activity, extracts were dried using nitrogen evaporation and analyzed by UHPLC-HRMS for identification.

### UHPLC-HRMS Profiling of QS-Active Supernatant Extracts

Samples were resuspended in approx. 150 μl 50:50 (vol/vol) acetonitrile (ACN)-water 50:50 (vol/vol) and 2 μl subsamples analyzed by UHPLC-HRMS. This was done on an Agilent Infinity 1290 UHPLC system (Agilent Technologies, Santa Clara, CA, United States) coupled to an Agilent 6550 QTOF MS operated in positive electrospray (ESI) mode, scanning *m/z* 50-1700. A lock mass solution of 10 μM Hexakis(2,2,3,3-tetrafluoropropoxy)phosphazene (Apollo Scientific Ltd., Cheshire, United Kingdom) dissolved in 95% acetonitrile and infused in the secondary ESI sprayer using an extra LC pump at a flow of 20 μl/min, and the [M + H]^+^ at m/z 922.0098 used as lock mass, resulting in a mass accuracy better than 3 ppm (deviation relative to theoretical m/z value).

An Agilent Poroshell 120 phenyl-hexyl column (2.1 × 150 mm, 2.7 μm), held at 60°C was used for separation. A linear gradient at 0.35 ml/min, consisting of water and ACN both buffered with 20 mM formic acid was started at 10% ACN and increased to 100% after 15 min, where it was held for 2 min. It was subsequently returned to 10% ACN in 0.1 min and maintained for 3 min (Kildgaard et al., 2014).

A reference standard mixture containing the following HSLs were included in the analytical sequence: C4, C6, C8, C10, C12, Oxo-C6, Oxo-C8, Oxo-C10, Oxo-C12, OH-C6, OH-C8, OH-C10, and OH-C12. Using the Agilent MassHunterQuant software, extracted ion chromatograms the [M + H]^+^ and [M + Na]^+^ ions ± 10 ppm were used for identification, along with the isotopic pattern (Kildgaard et al., 2014), and finally a retention time match ± 0.01 min.

### Growth Analysis of Marine Isolates

Isolates with distinct morphologies that were cultured on QS-treated plates and identified by 16S rRNA sequencing were grown on marine agar for 72 h at 23°C. Cells were transferred into fresh marine broth, OD_600 nm_ 0.05, in the presence and absence of 50 nM–10 μM of 3-oxo-C12 HSL, with DMSO as carrier control. Growth was measured spectrophotometrically at OD_600 nm_ in honeycomb plates incubated at 23°C on a Bioscreen-C automated growth curves analysis system (Growth Curves USA).

### Biofilm Assays

*Bacillus* sp. CH8a was grown in marine broth (MB) at 23°C overnight with shaking (Phelan et al., 2012). In order to monitor the impact on biofilm formation, 500 μl of cell-free supernatant (CFS) from the QS strain 3-day cultures was added to 500 μl of test cultures at OD_6__00 nm_ 0.1, grown in MB. As controls, 500 μl of fresh media was added to 500 μl of test cultures (media control) and 500 μl of the CFS of test cultures were added to 500 μl of corresponding test culture. In all cases, CFS was obtained by centrifugation at 8,000 rpm for 5 min followed by filtration through a 0.2 μm sterile filter. *Bacillus* sp. CH8a biofilms were incubated for 2 days at 23°C. Unattached cells were aspirated out of all wells which were washed once with 1 ml of sterile water. Attached cells were quantified using 0.1% (w/v) crystal violet. For marine isolate biofilm analysis, 3-oxo-C12-HSL was added at 10 μM to media and processed as described above.

### Co-culture Sponge Enrichment Assays

A simple co-culture system was designed to expose marine sponge homogenate to signals and metabolites from actively growing QS active isolates. Costar Spin-X 0.22 μm cellulose acetate centrifuge tube filters (Corning) consisting of an upper chamber with a filter base and a lower receptacle chamber was used for this purpose. QS active isolates were grown in nutrient media in the top chamber, while marine sponge homogenate was incubated in the lower chamber. While the marine sponge homogenate was physically separate from the QS active isolate at all times, transfer of small molecular signals and metabolites between both chambers was possible. In order to establish the validity of the co-culture system for QS transfer between the upper and lower chambers, as well as the integrity of the filter system to prevent bacterial leakage, several test studies were performed. The upper chamber was removed, and 1 ml of LB broth was added to the lower chamber. The upper chamber was replaced and a known QS producing organism *P. aeruginosa* PA14 (500 μl) was added. The tubes were incubated at 37°C for 24 h after which time the top chamber was removed. Aliquots of the lower chamber were tested for (a) activation of AHL biosensor strains *C. violaceum* and *S. marcescens* and (b) contaminating bacterial growth.

**FIGURE 1:**
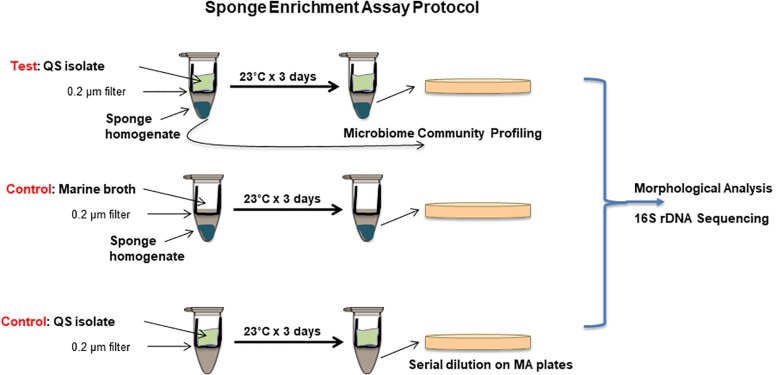
Co-culture sponge enrichment assay protocol incorporating QS and QQ samples with appropriate controls. QS or QQ producing isolates are placed in the top chamber with marine both media to facilitate growth. These are separated from the lower chamber containing sponge material by a 0.2 μm filter. QS or QQ signals can transition from the top chamber into the lower chamber by diffusion. Controls are included to ensure no microbial transfer between chambers. Isolation of culturable organisms is achieved on marine agar plates following 3 day incubation. Distinct morphologies are scored and selected for 16S rRNA sequencing.

Quorum sensing active isolates were grown in marine broth at 23°C for 72 h. *Stelletta normani* sponge sample (∼1 g) was homogenized in 10 ml of PBS. The upper chamber of the Costar Spin-X 0.22 μm cellulose acetate centrifuge tube filter (Corning) was removed, and 500 μl of sponge homogenate with 500 μl marine broth was added to the lower chamber of the filter tube. The filter was repositioned and 300 μl of QS active isolate in marine broth at OD_600 nm_ 0.2 was added. Several controls were included: (i) 300 μl of marine broth was added to the top chamber to provide a baseline profile of bacteria that could be cultured under the standard media conditions used, (ii) each QS active isolate was prepared as above with no sponge sample in the lower chamber to control for leakage through the membrane, and (iii) media controls were included to control for inadvertent contamination (Figure 1). The tubes were incubated at 23°C with gentle shaking at 50 rpm for 72 h at which time the upper chambers were carefully removed. The contents of the lower chamber were mixed gently and serially diluted. Dilutions were plated on marine agar and incubated at 23°C for at least 72 h. Colony numbers and morphologies were profiled and distinct isolates were identified by 16S rDNA sequencing.

### Draft Genome Sequencing of QS Active *Psychrobacter* sp.

Total DNA of Psychrobacter sp. 230 strain was obtained using the UltraClean microbial DNA isolation kit (Mo Bio Laboratories, Inc., Carlsbad, CA, United States) and was used for DNA library preparation using a TruSeq exome library prep kit. The draft genome sequencing project of Psychrobacter sp. 230 strain was performed by the Beijing Genomics Institute (BGI, China) using the Illumina HiSeq 4000 sequencing platform involving paired-end reads with a read length of 150 bp. The superfast FASTA/Q file manipulation tool, readfq.v5 (BGI, unpublished software), was used for quality trimming. This software removes the paired-end reads with a certain proportion of low-quality bases (default, 40%; parameter setting, 6 bp), reads with a certain proportion of Ns (ambiguous bases; default, 10%; parameter setting, 10 bp), reads with adapter contamination (default, 15 bp overlapped between adapter and reads), and duplicate sequences. Thus, the high-quality-filtered reads were all 150 bp long. From a total of 5.417.936 raw paired-end reads of 150 bp length, 3.329.182 high-quality reads were generated after processing with readfq.v5. Assembly was performed using SOAPdenovo 2.04 with default parameters. The sequencing depth provided 47.5 coverage of the genome. The draft genome assembly comprised 69 contigs with an N50 value of 183,949 grouped into 69 scaffolds with a total size of 3,290,930 bp and an overall GC content of 42.8%. The whole-genome shotgun project was deposited at NCBI under the accession number: NZ_SNVH00000000.1. The raw reads obtained after processing with readfq.v5 have been submitted to NCBI SRA under the accession number SRP216019.

### Phylogenetic Analysis

To infer the phylogenetic history, nucleotide sequences were retrieved and downloaded from NCBI: GenBank^2^. Sequences were aligned using Clustal Omega and were cut to consistent lengths (Sievers et al., 2011). The evolutionary analysis was conducted in MEGA X using the Neighbor-Joining method (Saitou and Nei, 1987; Kumar et al., 2018). The clustering was tested using Bootstrapping with 1,000 replicates (Felsenstein, 1985). The Tajima–Nei method was used to calculate the evolutionary distances (Tajima and Nei, 1984). There was a total of 1,459 positions in the final dataset.

## Results

### QS Signaling Potential Within Marine Sponge Microbiomes

**FIGURE 2:**
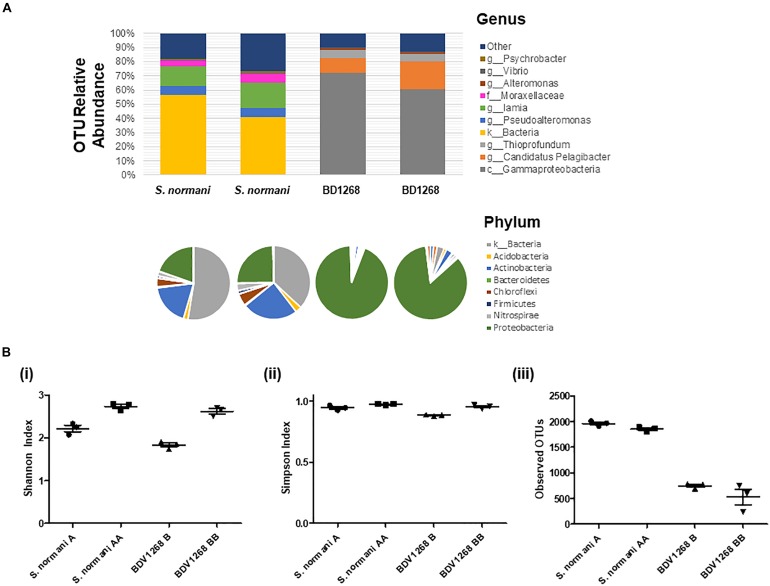
**(A)** Microbiome 16S DNA bacterial community profiling of sponge DNA. Stacked charts show relative abundance at the genus level. Phylum community profiles are presented in pie-chart format above the corresponding genus-level stacked chart. Comparison with culturable microbiota reveals considerable challenge in activating the “silent” majority of genera present. Two independent samples were taken for each sponge and the average of three independent DNA extraction sequenced replicates is presented for each sample. **(B)** Diversity and OTU analysis on microbiome data (i) Shannon diversity index (H = –Σ*p*_*i*_ ln *p*_*i*_), (ii) Simpson diversity index, and (iii) OTU abundance.

A range of QS active marine sponge microbial communities have been reported in recent years (Mohamed et al., 2008; Zan et al., 2012; Abbamondi et al., 2014; Britstein et al., 2018). The presence of QS systems in the sponge microbiota suggests a dynamic and ordered community that can respond to external cues and challenges. However, the extent to which QS producing bacteria colonize the marine sponge, and the role of QS within those microbial communities remains to be determined. Therefore, two distinct marine sponges were selected for microbial community profiling to establish the extent to which QS potential existed therein. Given the heterogeneity that exists in many clinical and ecological niches, with localized population profiles existing within relatively short distances of each other within a singular niche, two separate samples of each marine sponge species were selected. Homogenization and subsequent sequencing of two independently harvested triplicate samples revealed some interesting features of the respective microbiomes. Approximately 50% of the *S. normani* samples was identified only to the level of kingdom classification, suggesting a unique and unexplored bacterial diversity (Supplementary Table S1). The dominant phyla were Proteobacteria and Actinobacteria. At the genus level, *Iamia* [14.9% (±1.9)], *Pseudoalteromonas* [6.1% (±0.6)], *Moraxellaceae* [4.7% (±0.1)], and *Nitrospira* [2.5% (±0.6)] were the most abundant identifiable genera (Figure 2A). In contrast, the largest group identified for sponge BD1268 was the γ-Proteobacteria, representing between 85 and 94% of the OTUs in these samples. Sponge BD1268 was colonized by genera such as *Candidatus Pelagibacter* [12% (±4.5)], *Thioprofundum* [4.2% (±1.0)], and *Thiohalophilus* [2.4% (±0.4)]. Although the individual profiles remained relatively consistent from the perspective of the dominant phyla and families, there were differences in relative abundances between the microbiome profiles from the distinct sponge samples suggesting that heterogeneity of the population may exist within the sponge (Figure 2A). Diversity indexes (Shannon and Simpson) and OTU abundance was higher in the *S. normani* samples when compared with the respective samples from the BD1268 sponge (Figure 2B). As expected, independent samples clustered together based on sponge source (Supplementary Figure S1). Genera known to encode QS signaling systems (e.g., *Pseudomonas*, *Halomonas*, *Psychrobacter*) were present in the microbiomes of both sponges, although it was interesting to note that they were low in abundance when compared to the principal colonizers of the sponges. Therefore, notwithstanding the fact that genera previously shown to encode QS systems were present, the degree to which QS signaling pervades in these sponges remained to be determined.

### Identification of AHL Activities in Bacteria Isolated From Marine Sponges

The collection of morphologically diverse bacterial isolates from the *S. normani* sponge described above had previously been reported (Gutierrez-Barranquero et al., 2017). Together with this, cultivation of bacteria from sponge tissue derived from other sponge families such as *Hexactinellida*, *Lissodendoryx*, *Poecillastra*, *Inflatella*, and *Amphilectus* had yielded a collection of bacteria from the Proteobacteria, Actinobacteria, Bacteroidetes and Firmicutes groups (Gutierrez-Barranquero et al., 2017). Within these groups a wide range of different families were represented. The most abundant families included Pseudoalteromonadaceae, Flavobacteriaceae, and Moraxellaceae. To establish the prevalence of AHL based QS within the sponge microbiome, over 650 sponge isolated bacteria were screened for AHL production. In total 10 AHL producing candidates were identified, representing approximately 0.015% of the culture collection (Supplementary Figure S2 and Table 1). In order to identify the AHL positive isolates to a species level, the full length 16S rRNA (1,400 bp) sequence was determined. Among the isolates identified with AHL activity were a number of known AHL producers these included members of the *Halomonas*, *Psychrobacter*, *Vibrio, Pseudoaltermonas*, and *Pseudomonas* genera (Bruhn et al., 2005; Huang et al., 2009; Tahrioui et al., 2011; Ma et al., 2016). Despite the fact that the culture collection was populated by bacterial isolates harvested from six sponges, only four of these (*Stelletta*, *Hexactinellida*, *Inflatella*, and *Lissodendoryx*) yielded QS active isolates. The detection of QS active genera such as *Pseudomonas* and *Halomonas* from within the *S. normani* strains, despite the low abundance evident in the microbiome profile (Supplementary Table S1), is an important finding and suggests potentially a temporal role for QS active strains within communities. All QS active isolates were further validated by TLC analysis and soft agar biosensor overlay prior to selection for UHPLC-HRMS analysis and structural characterization (Supplementary Figure S2).

**TABLE 1:** AHL profile of marine sponge QS active bacterial isolates.

**Sample no**	**Sponge source**	**Biosensor activated-AHL**	**16S rRNA identification**	**UHPLC-HRMS verified QS profile**
12	*Lissodendoryx*	*S. marcescens* SP19	*Pseudomonas* sp.	ND
142	*Inflatella*	*S. marcescens* SP19	*Pseudomonas* sp.	O-C12
163	*Lissodendoryx*	*A. tumefaciens* NTL4	*Vibrio* sp.	O-C10, O-C12
230	*Hexactinellida*	*A. tumefaciens* NTL4	*Psychrobacter* sp.	O-C12
211	*Hexactinellida*	*A. tumefaciens* NTL4	*Pseudoalteromonas* sp.	O-C12
214	*Hexactinellida*	*A. tumefaciens* NTL4	*Pseudoalteromonas* sp.	ND
335	*Stelletta*	*C. violaceum* CV026 *A. tumefaciens* NTL4	*Halomonas* sp.	O-C10
394	*Hexactinellida*	*A. tumefaciens* NTL4	*Pseudoalteromonas* sp.	O-C12
411	*Stelletta*	*S. marcescens* SP19	*Pseudomonas* sp.	C4, C6^#^
419	*Stelletta*	*A. tumefaciens* NTL4	*Pseudoalteromonas* sp.	OH-C6^#^, OH-C10, O-C10, O-C12

*#Trace detected.*

To identify the AHLs being produced by each of the QS positive candidates UHPLC-HRMS was performed (Supplementary Figure S2). Ethyl acetate extracts were prepared for all isolates that had tested positive for AHL. All extracts were tested to ensure activation of respective AHL biosensors. Based on UHPLC-HRMS analysis, AHLs were identified in the majority of isolates (Table 1). The most abundant AHLs being produced by these isolates were 3-oxo-C10-HSL and 3-oxo-C12-HSL. Both AHLs, in addition to 3-OH-C10 HSL, were identified in extracts from *Psychrobacter* sp. and *Pseudoalteromonas* sp., the latter also producing trace amounts of 3-OH-C6 HSL. Only one isolate produced detectable amounts of C4-HSL, with the same *Pseudomonas* sp. isolate also producing trace amounts of C6-HSL. In some cases, no AHL traces were identified, notwithstanding the ability of the isolates to activate the respective biosensors.

### Co-culture With QS Positive Isolates Influences Culturable Outputs From Marine Sponge

**FIGURE 3:**
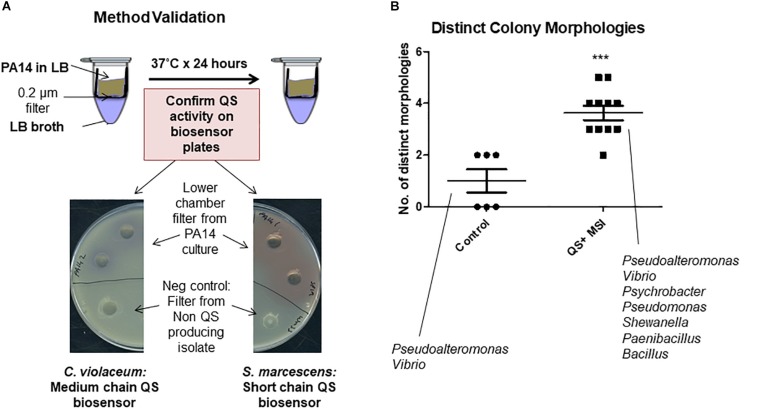
Co-culture sponge enrichment analysis. **(A)** Proof of concept using QS producing strains. Transfer of the QS active compounds confirmed by validation on Biosensor seeded plates. **(B)** Outcome of QS-mediated sponge enrichment assays measuring recoverable morphologies which were subsequently 16S rRNA-typed. Each datapoint refers to the number of distinct species recovered after the designated treatment. The species listed represent recoverable isolates identified in the study. Data presented represents at least three independent assays encompassing distinct *S. normani* sponge preparations. Statistical analysis was performed by Student’s *t*-test. ^∗∗∗^*p* ≤ 0.001.

The marine sponges profiled in this study appear to sustain a significant network of QS systems which may be important in modulating the population in response to external cues. Previous studies have shown that the ratio of QS and QQ strains can change dramatically in response to environmental conditions (Tan et al., 2015). Therefore, we investigated whether the secretome of the QS active isolates could impact on the profile of culturable bacteria that could be obtained from marine sponges under the standard media conditions used. Filter based microtubes were used to establish a co-culture system whereby the QS active isolate was added to a well with a porous membrane through which secreted QS signals can be transferred to the chamber below containing *S. normani* sponge homogenate (Figure 3A). In three independent studies, distinct colony morphologies were observed on plates with QS treated samples when compared to untreated controls (Figure 3B). The fidelity of all controls was maintained throughout the experiments with no leakage or inadvertent contamination observed.

*Pseudoalteromonas* and *Vibrio* were both cultured from the *S. normani* sponge homogenate in the absence of any treatments. Upon co-culture with QS active isolates, *Pseudomonas*, *Paenibacillus* and *Psychrobacter* were isolated in independent experiments. This was in addition to *Pseudoalteromonas* and *Vibrio* as seen on the control plates. Co-culture with QS-active isolates 12 and 411 resulted in isolation of *Pseudomonas* while 214 and 211 led to isolation of *Paenibacillus*. *Psychrobacter* was isolated upon co-culture with all 211, 214, and 411. No representative from these genera was observed on the untreated control plates (Figure 3). All three genera were represented at a very low relative abundance within the microbiome from the untreated *S. normani* sponge samples (*Pseudomonas* [0.003% (±0.003), *Psychrobacter* [0.6% (±0.1)], and *Paenibacillus* only evident at Order level of *Bacillales* [0.02% (±0.05)]) suggesting a change in community structure (Supplementary Table S1). Together, these data suggest that cell-cell communication and QS can impact the dynamics of population growth within microbiomes and influence the culture-readiness of genera in response to external cues. It was notable that the impact of QS active supernatants was not restricted to gram negative organisms. However, the vast majority of microbiome constituents were not represented in the culturable diversity on the plates. Representatives of e.g., *Iamia*, *Nitrospira*, *Caldilinea*, or *Gaiella* were not observed on either control or test plates. Therefore, modification of the media to cater for additional supplemental requirements, or indeed to reduce nutritional richness, may be required to capture these unculturable components.

### QS Phenotype Profiling

**FIGURE 4:**
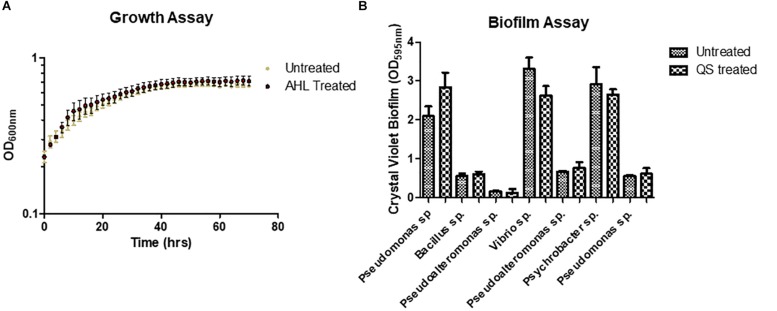
Addition of purified 3-oxo-C12-HSL did not influence **(A)** growth of a *Pseudomonas* sp. isolate or **(B)** biofilm formation of marine sponge isolates recovered in this study following co-culture.

In order to understand how QS signaling might influence species dynamics within the marine sponge, we investigated whether marine isolates obtained following co-culture with QS positive marine sponge isolates responded to exogenous AHLs. As the most frequently identified AHL in this study, 3-oxo-C12 HSL was selected to assess its influence on two key QS associated phenotypes i.e., biofilm formation and growth. Somewhat surprisingly, neither was affected upon addition of 3-oxo-C12 HSL when compared to untreated samples (Figure 4). Addition of either 10 or 50 μM 3-oxo-C12 HSL did not impact on growth of test strains, either in exponential or stationary phase, as determined over 72 h (Figure 4 and Supplementary Figure S3). Similarly, although the test isolates formed biofilms in multi-well plates, addition of either 10 or 50 μM 3-oxo-C12 HSL did not significantly alter total attached biomass (Figure 4).

**FIGURE 5:**
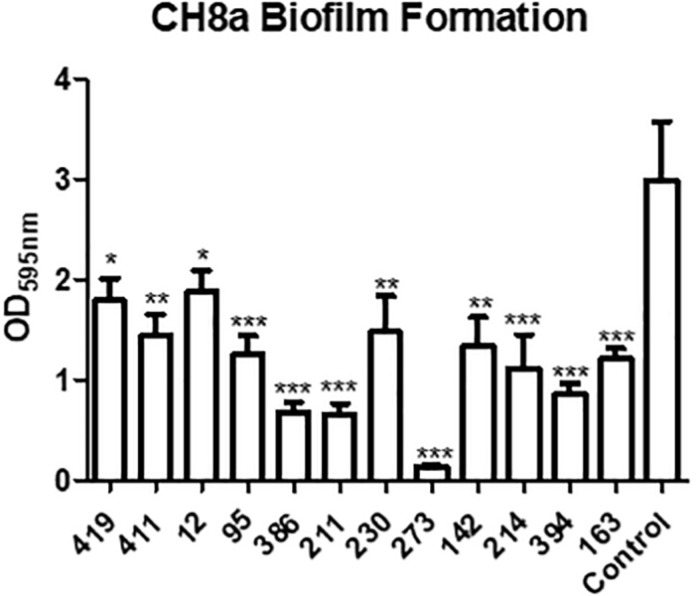
Modulation of *Bacillus* sp. CH8a biofilm formation by extracts from QS active isolates. Data presented is the average of at least three independent biological replicates. Statistical analysis was performed by one-way ANOVA with *post hoc* Bonferroni corrective testing (^∗^*p* ≤ 0.05, ^∗∗^*p* ≤ 0.005, ^∗∗∗^*p* ≤ 0.001).

The ability of QS to control a range of virulence related phenotypes is well established. In addition to controlling biofilm formation and toxin secretion in important pathogens, QS is also known to elicit an antagonistic response toward co-colonizing organisms in producing strains. To test if this applied to the marine sponge QS active isolates, biofilm formation in the marine sponge sporefomer *Bacillus* sp. CH8a was investigated in the presence and absence of extracts from QS positive isolates. Addition of extracts from several of the isolates led to a significant reduction in biofilm formation by CH8a when compared with the untreated control (Figure 5). This suggests that QS active strains would likely produce compound(s) that would moderate the behavior of co-existing microbes within the sponge microbiome.

### Genome Sequencing and Identification of LuxR Domain Proteins Within the *Psychrobacter* Genus

Suppression of biofilm and activation of QS biosensors suggest that QS signaling is a highly networked and evolved system in the marine sponge niche. However, very little is known about the factors involved in newly emerging QS positive species. *Psychrobacter* sp. have only recently been shown to be QS positive, although the molecular mechanism underpinning this activity remains to be elucidated. To assess if any proteins encoded within *Psychrobacter* genomes could function as LuxR homologs the available *Psychrobacter* genomes were investigated via the Pfam Domain Search Tool to identify proteins with specific domains associated with LuxR type transcriptional regulators, i.e., the autoinducer binding domain (PFAM03472) or the LuxR DNA binding domain (PFAM00196). A total of 70 proteins were identified in 38 genomes that possessed a LuxR type DNA binding domain (Supplementary Table S1). However, neither an autoinducer binding domain nor a classical autoinducer synthase domain (PFAM00765) were identified in any of the available genome sequences. This raised two possibilities; (i) a potential novel AHL synthase with a low sequence homology to known AHL synthases may be functionally active within the *Psychrobacter* genus, or (ii) marine *Psychrobacter* sp. encode novel regions of DNA carrying the capacity for AHL production. Several reports exist in the literature of QS active strains where the corresponding genetic systems encoding that activity remain to be identified. The prevalence of horizontal gene transfer, and the phenotypic and genotypic heterogeneity that exists within communities is such that interrogating model genomes can be limited when searching for a particular functionality that may be strain specific. We considered it important to investigate the QS signaling potential that is encoded in the genome of the *Psychrobacter* species, particularly as we had a QS active strain with which to interrogate.

**FIGURE 6:**
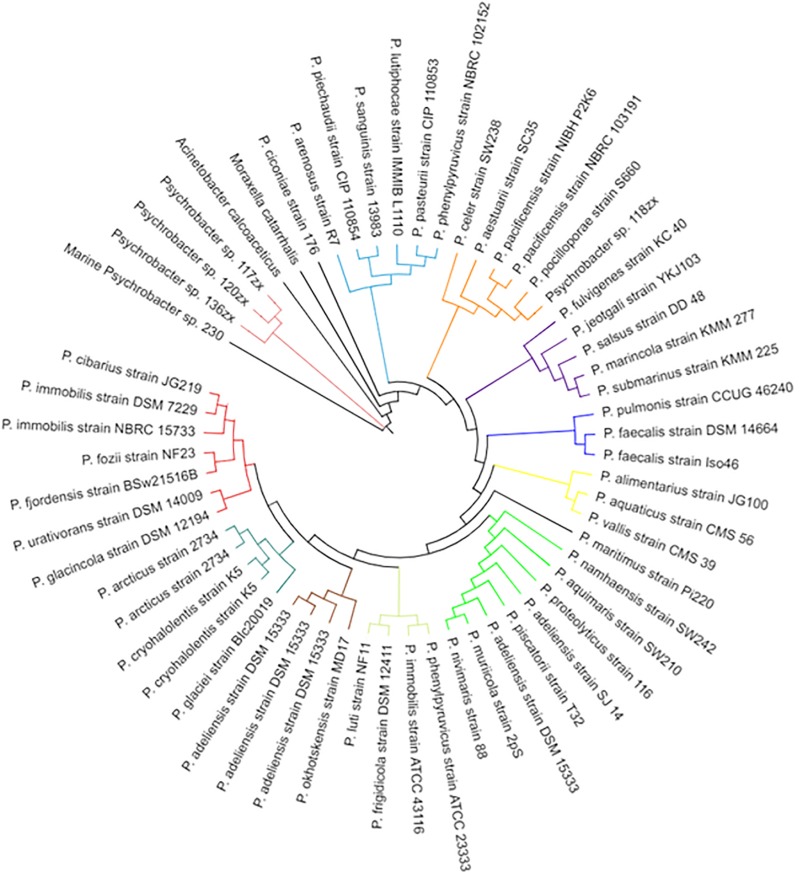
Phylogenetic tree of *Psychrobacter* species including the marine *Psychrobacter* sp. 230 isolate identified in this study. Species names are listed on the outer edge of each branch and an arbitrary color based system is used to distinguish the various clades in the tree.

Whole genome sequencing of the *Psychrobacter* sp. 230 isolate from this study revealed a genome encoding 2908 genes (Supplementary Figure S4). The draft genome assembly comprised 69 contigs with an N50 value of 183,949 grouped into 69 scaffolds with a total size of 3,290,930 bp and an overall GC content of 42.8% (Table 2). The *Psychrobacter* sp. 230 genome was searched for Lux domains and three putative LuxR domain proteins were identified. BLASTX sequence searches and SMART domain analysis suggested that these three proteins were transcriptional response regulator proteins, with no evidence of autoinducer domains. A LysE family homoserine(lactone) translocation protein was also encoded in the genome, as was a dienelactone hydrolase family protein. However, the absence of a LuxI-like synthase gene in the *Psychrobacter* genome indicates that the molecular mechanism through which AHL based QS is performed in this isolate remains to be ascertained. Cluster based analysis revealed that this *Pscyhrobacter* sp. grouped with three other *Psychrobacter* sp. isolates and distinct from other members of the genus suggesting it may be a genetic outlier within *Psychrobacter* (Figure 6). Previously, Ma et al. (2016) identified a *Psychrobacter* sp. isolate from mangrove with QS activity that clustered with *Psychrobacter* sp. isolates from deep sea sediments of the east Pacific Ocean. Therefore, a functional approach may be warranted to uncover the molecular basis of QS signaling in this species.

**TABLE 2:** Genome data for *Psychrobacter* sp. 230 marine sponge isolate.

***Psychrobacter* sp. 230**	
Genome size	3,290,930
Contigs (*n*)	1
GC content (%)	42.8
Protein coding genes	2857
RNA genes	44

## Discussion

In this study, 650 marine sponge bacterial isolates were screened for the ability to produce AHLs. A total of 10 isolates were identified that were capable of activating AHL biosensor reporter strains. Mass spectrometry revealed that several of the isolates produced the same or similar AHLs (OC10–OC12 HSL). The capacity for AHL based signaling in the marine ecosystem has previously been reported. AHL signaling in marine snow was first described by Gram et al. (2002), with species of *Roseobacter* shown to be QS active. More recently, *Pantoea ananatis* has been reported to produce a spectrum of AHL signals in marine snow, governing extracellular enzyme production in producing strains (Jatt et al., 2015). Since the first description of AHL based QS in *A. fischeri* species (Nealson and Hastings, 1979), where the LuxIR paradigm system was first identified, AHL signals have been found in a broad diversity of marine isolates (Hmelo, 2017). Rasch et al. (2007) described AHL production in *Aeromonas salmonicida* isolates, while a number of studies profiled members of the Vibrionaceae for AHL production (Yang et al., 2011; Purohit et al., 2013). The diversity of AHL signals that are encoded in the marine ecosystem has been highlighted by a recent study reporting AHLs with long (up to 19 carbons) and poly-hydroxylated acyl side chains (Doberva et al., 2017). At the same time, studies reporting QS inhibition or quenching in the marine environment have also received considerable attention in recent years (Romero et al., 2012; Gutierrez-Barranquero et al., 2017; Ma et al., 2018). Primarily produced by microbial species, host derived quorum quenching (QQ) has also been described (Weiland-Brauer et al., 2019). Elucidating and profiling the extent of QS signaling within these environments is a key step in understanding the functional role played by QS in the host-microbe interaction.

Interspecies communication within the microbial communities of the marine sponge may offer a competitive advantage through cross-genus coordinated behavior. If several species all produce the same or similar AHLs, then they potentially could adopt community like behaviors more rapidly than species that are not part of this interspecies signaling network. This could arise from the activity threshold being reached more rapidly if several different species produce the same signaling molecule. Of course, conservation within receptor systems would also be an integral factor in moderating these responses. The prevalence of orphan LuxR receptor systems in sequenced microbial genomes highlights the complexity of signaling interactions that remain to be identified and understood (Patankar and Gonzalez, 2009). Adopting community-like behaviors through QS systems may offer a distinct competitive advantage as bacteria can attach to a form a biofilm like structure within the environment of the sponge. Community-based small molecular interactions may also be important with respect to intracellular sponge symbionts, such as the recently reported *Candidatus* Endohaliclona renieramycinifaciens intracellular interaction with *Haliclona* (Tianero et al., 2019).

The prevalence and diversity of AHLs being produced by the sponge bacterial isolates identified in this study suggests that mechanisms to inhibit these systems may also exist within the sponge microenvironment. The identification of novel compounds that are capable of inhibiting AHL based QS systems is one of the key areas of focus in the development of next generation antimicrobials. Previously, we have reported on the profiling of a subset of this collection of marine sponge isolates for quorum sensing inhibitory (QSI) or QQ activity. A total of 18/440 culturable isolates were found to encode QSI, being able to supress AHL signaling is an isolate dependent manner (Gutierrez-Barranquero et al., 2017). It was interesting to note in that study that several species possessed dual QS and QSI activities. In this current study the finding that *Psychrobacter* sp. isolates from the same sponge collection were also capable of QS activity suggests that community level moderation of group behavior is a highly evolved trait in the marine ecosystem. Tan et al. (2015) previously showed how the dynamics of QS and QSI/QQ producing organisms can fluctuate in response to changes in environmental conditions. It is noticeable in this regard that two species cultured from QS treated sponge homogenate, *Pseudomonas* and *Paenibacillus*, are themselves known to possess AHL signaling systems (Ma et al., 2016). Understanding the interplay between QS and QSI/QQ in the marine sponge ecosystem and the role of QQ in moderating community behavior will underpin advances in marine ecology and beyond.

The dynamics of AHL production in marine microbial communities is seen as a mechanism to enhance culturability of rare genera, many of which encode valuable biosynthetic gene clusters for natural products such as antibiotics and anti-cancer drugs (Reen et al., 2015). While co-culture with QS positive isolates did alter the profile of culturable bacteria isolated from marine sponge homogenates, they failed to introduce new genera into culture. This of course could be due to limitations in the culture conditions, including the general nature of the media used which is more conducive to the culture of fast-growing bacteria. Dilution based methods and modification of the growth conditions with regard to media, temperature, and time may provide the optimum conditions for culture of QS dependent organisms (Rygaard et al., 2017).

The absence of a LuxIR system in the QS positive *Pychrobacter* sp. 230 isolate would suggest that a hidden diversity to the molecular mechanisms underpinning QS signaling remains to be elucidated. This is consistent with previous reports of AinS and LuxM family autoinducer synthase enoding genes, quite distinct from their LuxI counterparts (Venturi and Subramoni, 2009). Recently, a new LuxIR based system termed TswIR has been identified in an uncultured symbiont from the Red Sea Sponge *Theonella swinhoei* (Britstein et al., 2016). The synthase protein TswI (COG3916) was annotated as both an autoinducer synthase and a GNAT acetyltransferase activity and while GNAT acetyltransferase proteins were identified in the *Psychrobacter* genomes, no members of the COG3916 family were found. Furthermore, the recent finding that LuxIR homologs can synthesize and respond to non-acyl HSL signals, serves to underscore the hidden complexity in these systems (Ahlgren et al., 2011). Two orphan Photorhabdus LuxR proteins, PluR and PauR, sense alpha-pyrones and dialkylresorcinols, respectively (Brameyer and Heermann, 2015). It is possible that other examples of non-AHL LuxR interactions may be uncovered in the future, something that would add greatly to the complexity of the signaling interactions as currently understood. The absence of homologs of these proteins in the *Psychrobacter* sp. 230 genome may necessitate a functional approach in order to elucidate the molecular mechanism through which AHL signaling is established in this and other marine genera.

## Data Availability

The datasets generated for this study can be found in the NCBI Database accession nos: MN209943-MN209952, NZ_SNVH00000000.1, SRP216019 and PRJNA555824.

## Author Contributions

FR and FO’G conceived the study. FR, JG-B, CA, DW, RM, SS, and KN performed the experimental analysis. FR wrote the manuscript with inputs from all the authors. FR and FO’G finalized the manuscript for submission.

## Conflict of Interest Statement

The authors declare that the research was conducted in the absence of any commercial or financial relationships that could be construed as a potential conflict of interest.

## Footnotes

1 http://qiime.org/

2 http://www.ncbi.nlm.nih.gov/

## Supplementary Material

FIGURE S1 PCA bi-plot cluster analysis of microbiome samples performed using R (v. 3.5.2). Visualization was performed using the ggplot and ggfortify packages. Samples from both sponges form separate clusters reflecting their distinct microbial community profiles.

FIGURE S2 **(A)** Screen of Marine Sponge Isolates using QS-Biosensor Strains. **(B)** QS positive isolates were grown in culture flasks to confirm biosensor activation. Species level identification was achieved by 16S rRNA sequencing and subsequent BLAST analysis. **(C)** Extracts were validated by TLC overlay and subsequently sent for UHPLC-HRMS analysis and classification.

FIGURE S3 **(A–F)** Growth profiling of isolates harvested following co-culture with QS active strains in the presence of 10 μM 3-oxo-C12-HSL or DMSO carrier control. Data presented is the average (±SEM) of two independent biological replicates with five technical replicates in each experiment performed on the BioScreen-C.

FIGURE S4 Genome representation of the newly sequenced *Psychrobacter* sp. 230 isolate identified as a 3-oxo-C12-HSL producer in this study. COG functional categories are presented on the outer ring, while forward and reverse strand gene annotations are presented in the inner rings in red and blue, respectively.
