# Supplementary figures and images for: Quorum Sensing Signaling Alters Virulence Potential and Population Dynamics in Complex Microbiome-Host Interactomes
Source: Front Microbiol. 2019 Sep 11;10:2131. doi: 10.3389/fmicb.2019.02131 (PMC6749037; doi:10.3389/fmicb.2019.02131)

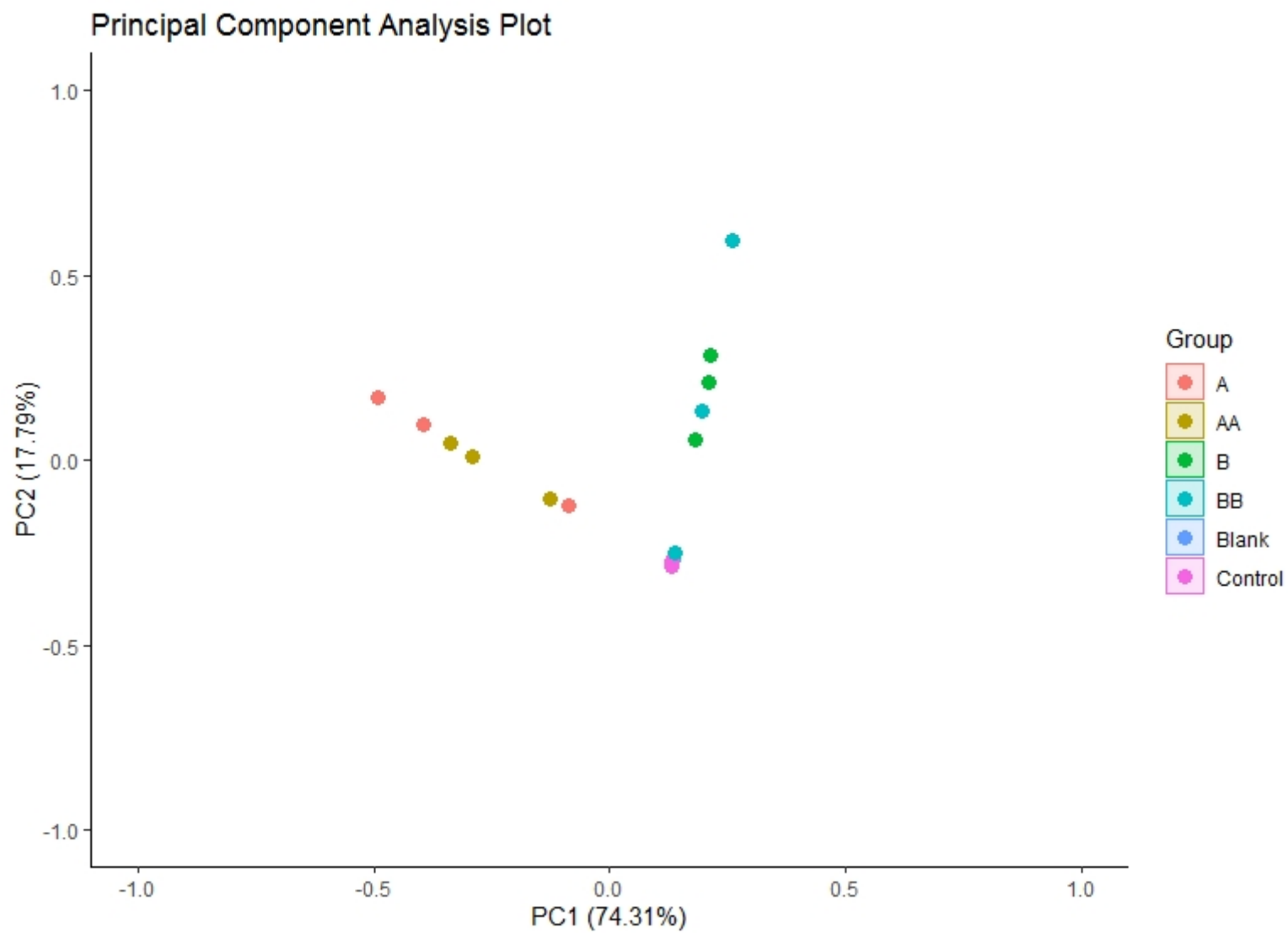

Supplementary Figure S1

Supplement: FIGURE S1 — PCA bi-plot cluster analysis of microbiome samples performed using R (v. 3.5.2). Visualization was performed using the ggplot and ggfortify packages. Samples from both sponges form separate clusters reflecting their distinct microbial community profiles. [file Data_Sheet_1.PDF]

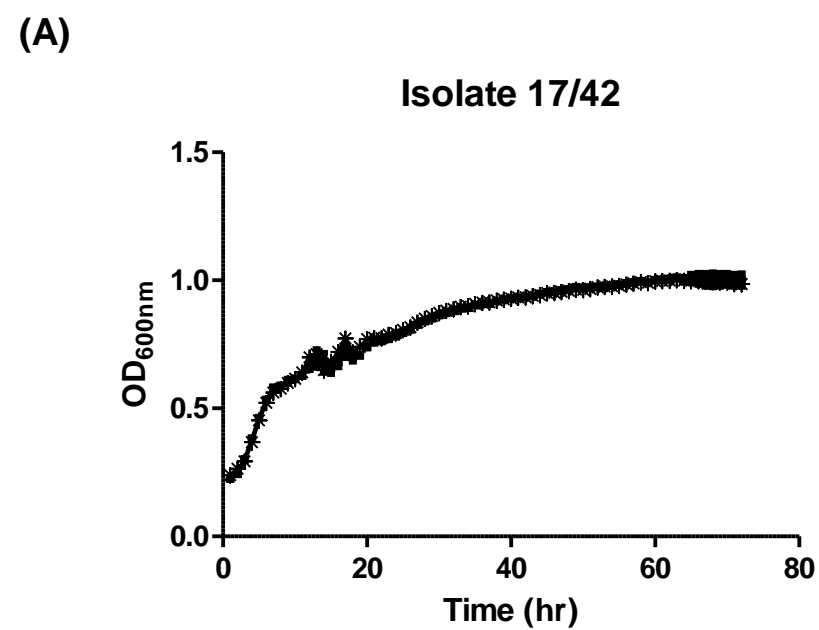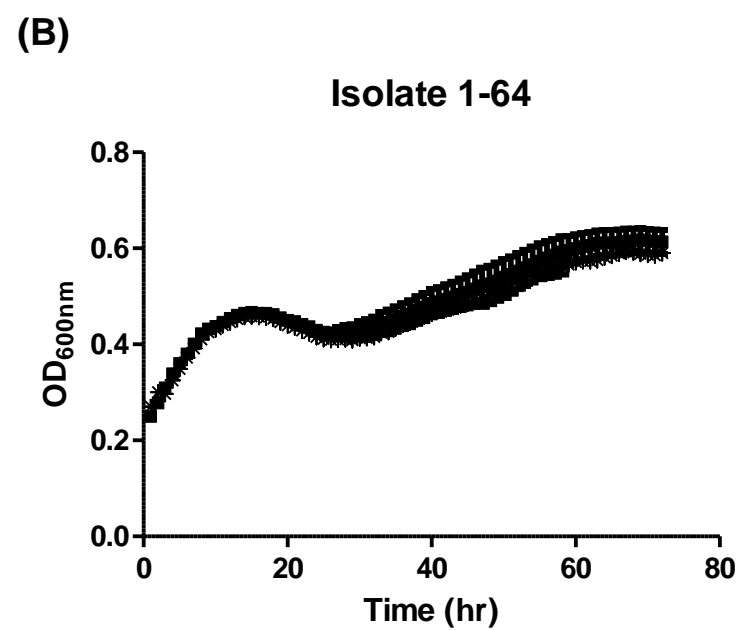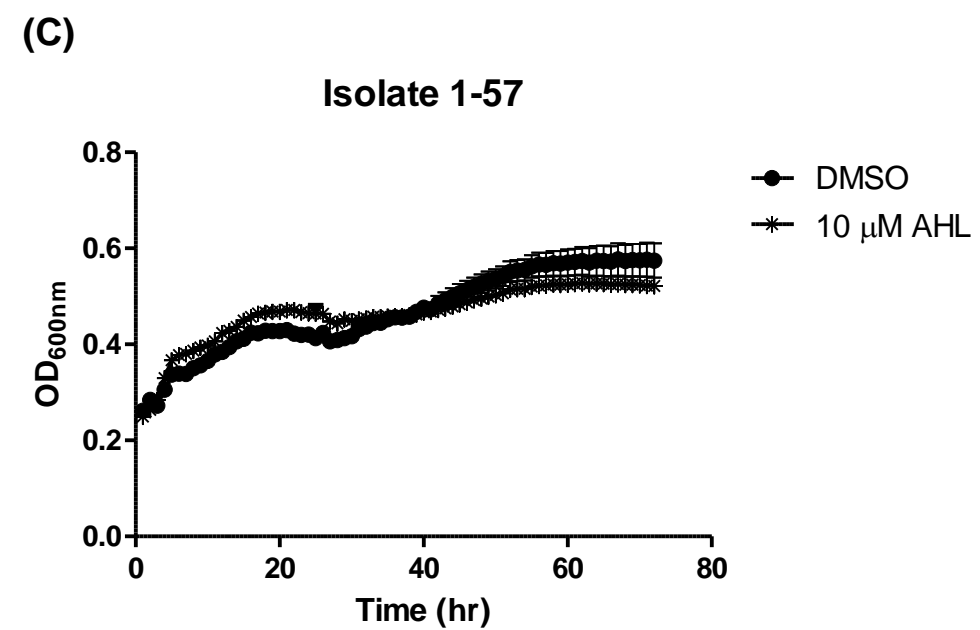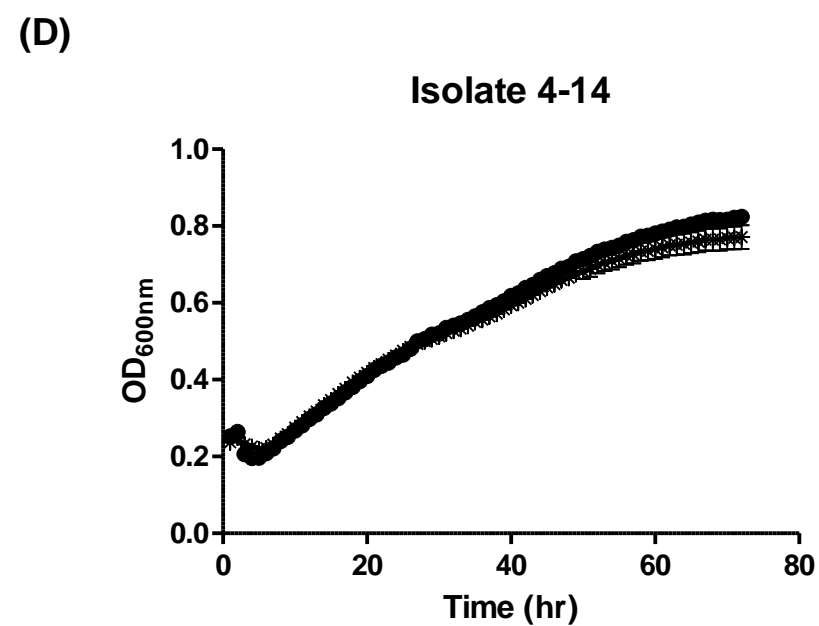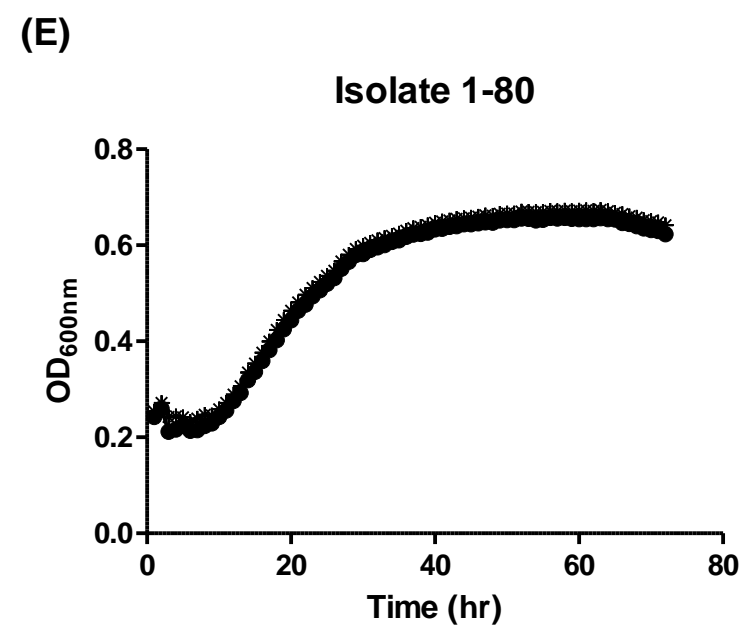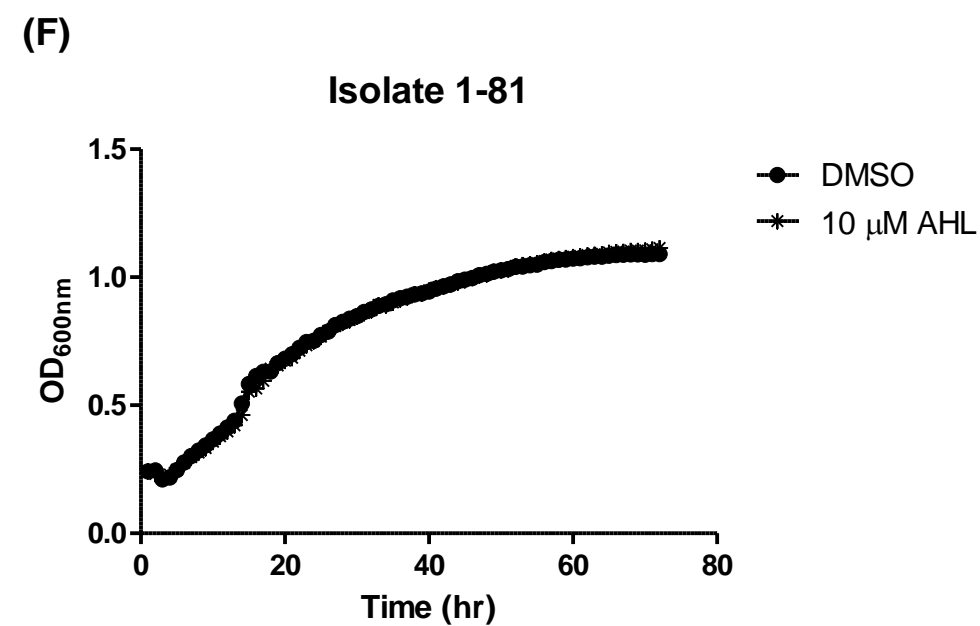

Supplementary Figure S3

Supplement: FIGURE S3 — (A–F) Growth profiling of isolates harvested following co-culture with QS active strains in the presence of 10 μM 3-oxo-C12-HSL or DMSO carrier control. Data presented is the average (±SEM) of two independent biological replicates with five technical replicates in each experiment performed on the BioScreen-C. [file Data_Sheet_3.PDF]
