# Supplementary material for: Quorum Sensing Signaling Alters Virulence Potential and Population Dynamics in Complex Microbiome-Host Interactomes
Source: Front Microbiol. 2019 Sep 11;10:2131. doi: 10.3389/fmicb.2019.02131 (PMC6749037; doi:10.3389/fmicb.2019.02131)
Supplement: FIGURE S2 — (A) Screen of Marine Sponge Isolates using QS-Biosensor Strains. (B) QS positive isolates were grown in culture flasks to confirm biosensor activation. Species level identification was achieved by 16S rRNA sequencing and subsequent BLAST analysis. (C) Extracts were validated by TLC overlay and subsequently sent for UHPLC-HRMS analysis and classification. [file Data_Sheet_2.PDF]

(A)

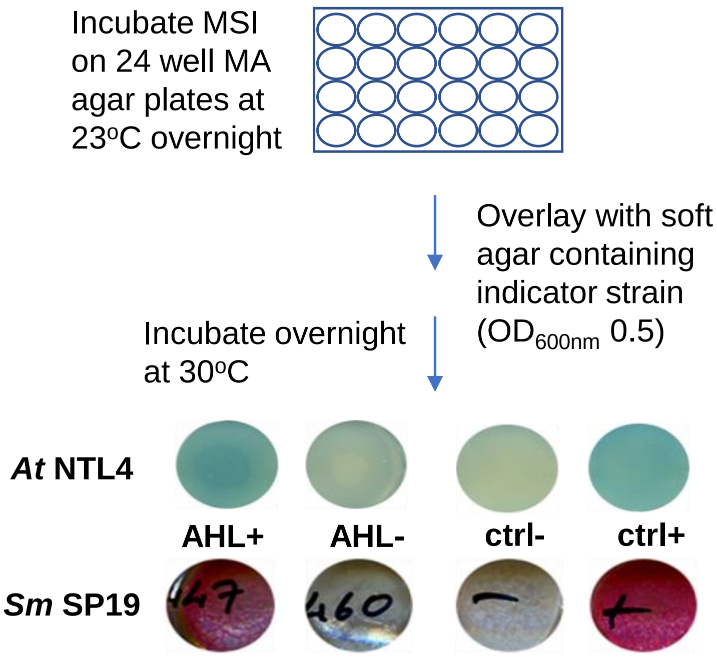

(B)

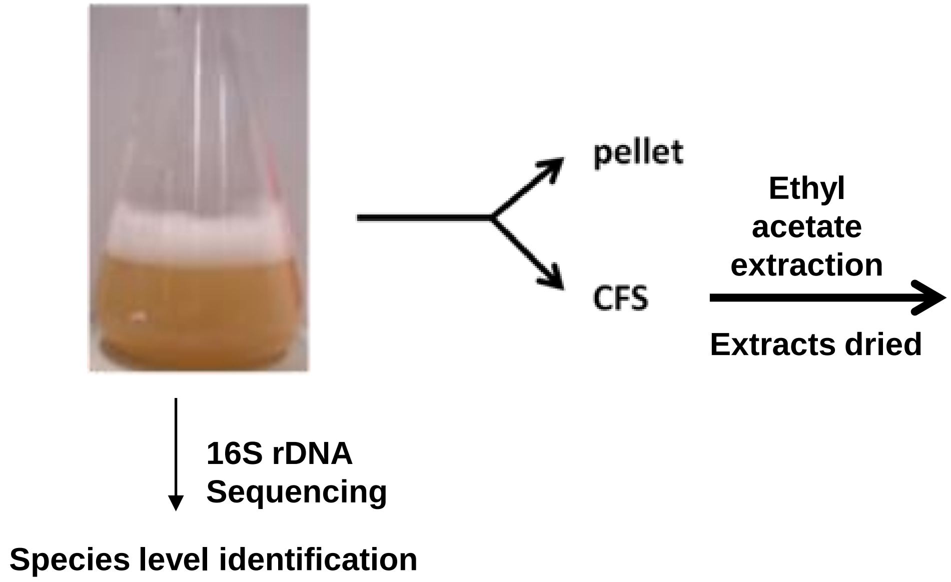

(C)

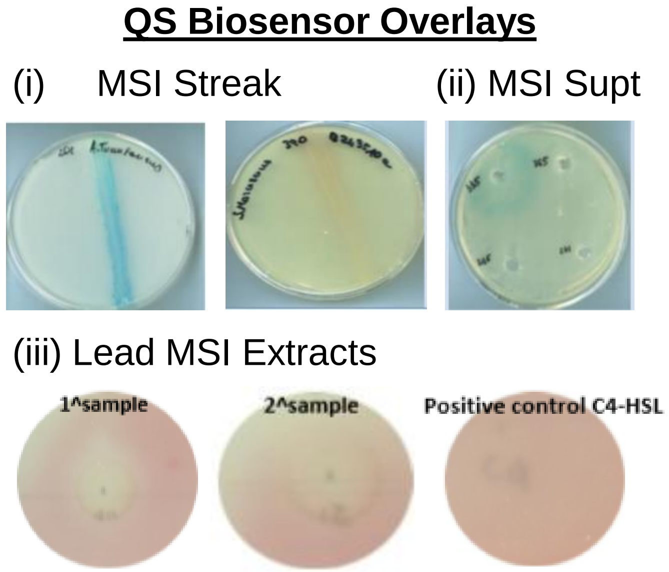

Sample No	Biosensor Activated	Isolate Classification	TLC Spot Assay (Size mm)
12	*S.marcescens SP19*	*Pseudomonadaceae*	13
142	*S.marcescens SP19*	*Pseudomonadaceae*	5
163	*A.tumefaciens NTL4*	*Vibrionaceae*	6
230	*A.tumefaciens NTL4*	*Moraxellaceae*	6
211	*A.tumefaciens NTL4*	*Pseudoalteromonadaceae*	10
214	*A.tumefaciens NTL4*	*Pseudoalteromonadaceae*	6
335	*A.tumefaciens NTL4*	*Halomonadaceae*	8
394	*A.tumefaciens NTL4*	*Pseudoalteromonadaceae*	6
411	*S.marcescens SP19*	*Pseudomonadaceae*	30
419	*A.tumefaciens NTL4*	*Pseudoalteromonadaceae*	6

Supplementary Figure S2
