# Supplementary material for: Quorum Sensing Signaling Alters Virulence Potential and Population Dynamics in Complex Microbiome-Host Interactomes
Source: Front Microbiol. 2019 Sep 11;10:2131. doi: 10.3389/fmicb.2019.02131 (PMC6749037; doi:10.3389/fmicb.2019.02131)
Supplement: FIGURE S4 — Genome representation of the newly sequenced Psychrobacter sp. 230 isolate identified as a 3-oxo-C12-HSL producer in this study. COG functional categories are presented on the outer ring, while forward and reverse strand gene annotations are presented in the inner rings in red and blue, respectively. [file Data_Sheet_4.PDF]

Psychrobacter sp. 230 Genome

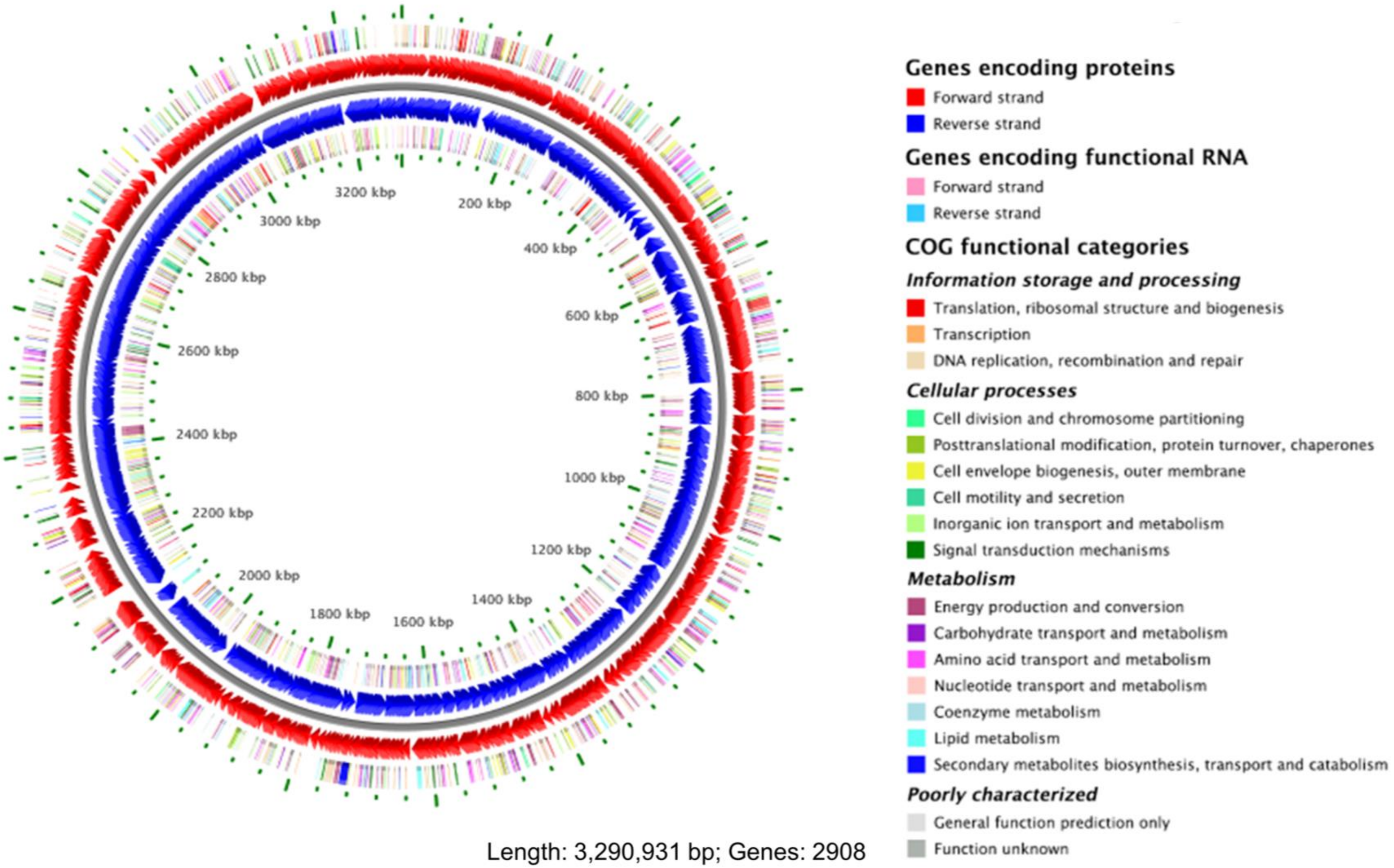

Supplementary Figure S4
